# Brief Engagement and Acceptance Coaching for Community and Hospice Settings (the BEACHeS Study): Protocol for the development and pilot testing of an evidence-based psychological intervention to enhance wellbeing and aid transition into palliative care

**DOI:** 10.1186/s40814-019-0488-4

**Published:** 2019-08-20

**Authors:** Nicholas J. Hulbert-Williams, Sabrina Norwood, David Gillanders, Anne Finucane, Juliet Spiller, Jenny Strachan, Sue Millington, Brooke Swash

**Affiliations:** 1 Centre for Contextual Behavioural Science, School of Psychology, University of Chester, Parkgate Road, Chester, CH1 4BJ UK; 2 School of Health in Social Science, University of Edinburgh, Edinburgh, UK; 3 Marie Curie Hospice Edinburgh, Edinburgh, UK

**Keywords:** Palliative care, Acceptance and Commitment Therapy, Cancer, Single-case design

## Abstract

**Background:**

Cancer affects millions of individuals globally, with a mortality rate of over eight million people annually. Although palliative care is often provided outside of specialist services, many people require, at some point in their illness journey, support from specialist palliative care services, for example, those provided in hospice settings. This transition can be a time of uncertainty and fear, and there is a need for effective interventions to meet the psychological and supportive care needs of people with cancer that cannot be cured. Whilst Acceptance and Commitment Therapy (ACT) has been shown to be effective across diverse health problems, robust evidence for its effectiveness in palliative cancer populations is not extensive.

**Method:**

This mixed-methods study uses a single-case experimental design with embedded qualitative interviews to pilot test a novel intervention for this patient group. Between 14 and 20 patients will be recruited from two hospices in England and Scotland. Participants will receive five face-to-face manualised sessions with a psychological therapist. Sessions are structured around teaching core ACT skills (openness, awareness and engagement) as a way to deal effectively with challenges of transition into specialist palliative care services. Outcome measures include cancer-specific quality of life (primary outcome) and distress (secondary outcome), which are assessed alongside measures of psychological flexibility. Daily diary outcome assessments will be taken for key measures, alongside more detailed weekly self-report, through baseline, intervention and 1-month follow-up phases. After follow-up, participants will be invited to take part in a qualitative interview to understand their experience of taking part and acceptability and perceived effectiveness of the intervention and its components.

**Discussion:**

This study is the first investigation of using ACT with terminally ill patients at the beginning of their transition into palliative treatment. Using in-depth single-case approaches, we will refine and manualise intervention content by the close of the study for use in follow-up research trials. Our long-term goal is then to test the intervention as delivered by non-psychologist specialist palliative care practitioners thus broadening the potential relevance of the approach.

**Trial registration:**

Open Science Framework, 46033. Registered 19 April 2018.

## Background

Globally, over 14 million new cases of cancer are diagnosed each year, and 8.4 million deaths were attributed to cancer in 2012 alone [1]. Finding out that cancer is no longer curable can be psychologically distressing for both patients and their family [2]. Indeed, recognition of distress as the sixth ‘vital’ sign to monitor in cancer care is receiving increased attention [3, 4]. The subsequent transition following a referral into specialist palliative care services when cancer is no longer curable can be a time of uncertainty and fear. When living with uncertainty, it can become difficult to satisfactorily plan for the future [5] which is crucial at this stage of cancer [6]. Quality of life may also be negatively affected [7]. There is, therefore, a clear need for effective interventions to meet the psychological and supportive care needs of cancer patients at this point in their illness [8] which the current evidence base does not provide [9].

Acceptance and Commitment Therapy (ACT [10]) is a form of cognitive behavioural therapy that has a good evidence base for the type of psychological problems often reported by people affected by cancer, for example, anxiety and depression [11], but specific evidence for its effectiveness in palliative cancer populations is lacking/limited [12]. Within the ACT model, distress is understood as being a normal reaction to a difficult situation, and ACT supports people to become more resilient [13] and self-compassionate [14] when in distress. ACT is underpinned by a number of therapeutic processes, including mindfulness, acceptance and cognitive defusion [15], which then support the individual to be more engaged with values-based living [12]. Within a palliative care setting, this framework is suggested as a helpful approach in supporting people to identify what is important to them and to help them live a life of meaning, quality and value, even when faced with challenging circumstances. Overall, ACT encourages people to be psychologically flexible [16], which is thought may help with distress management in people with incurable cancer [17].

A number of cross-sectional studies have demonstrated associations between ACT therapeutic processes, distress and quality of life outcomes in cancer patients [18, 19]. Other work has shown that psychological flexibility correlates also with positive adjustment indicators, such as benefit finding [20]. Intervention studies have reported feasibility and acceptability of ACT self-help interventions [21], and there is early evidence for the effectiveness of interventions in both curative and advanced cancer samples [22].

Whilst there are sound theoretical reasons why ACT should be useful in palliative care settings [12], we are aware of just one study which is feasibility testing the use of ACT for patients who are more established within the palliative care pathway [17]. Research is therefore needed to establish its broader empirical support [9]. In addition, although there have been recent studies exploring mechanisms and moderators of ACT interventions (e.g. increases in psychological flexibility and reduction in experiential avoidance) [23–25], these mechanisms, and how they relate to specific therapeutic components, are not well understood. Importantly, these processes have not yet been thoroughly explored in the context of a terminal cancer diagnosis, a setting which brings unique personal and environmental challenges.

This work aims to develop, and pilot test, a brief, manualised psychological intervention to provide support to people with an incurable cancer diagnosis who are at the transition into specialist palliative care services. We aim to further explore the feasibility of delivering this intervention within a hospice and community environment. Our planned study allows us to tentatively test the effectiveness of the intervention in a small sample of participants using a single-case, controlled design. We will explore mechanisms and processes of improvement in patient wellbeing through intervention delivery and short-term follow-up to further refine the intervention for subsequent research and clinical use. We are also monitoring data on acceptability, recruitment, attrition and eligibility in order to determine best practices moving forward into larger trials.

## Methods/design

This study uses a mixed-methods approach to both design and pilot test a novel psychological intervention for this patient group. A mixed-methods approach is appropriate for this research question as quantitative data can be supplemented by ideographic narrative data that records the unique perspective of patients in the trial [26]. Mixed-methods also allow researchers to evaluate the saliency and acceptability of defined constructs and components during a pilot test [27], ensuring suitability prior to larger, more expensive trials. We will incorporate patient feedback through the full development process (at design, intervention development and evaluation) to ensure that the intervention is acceptable to the patient group for whom it is designed [28].

The intervention will be delivered individually, using a single-case experimental design [29], to community-based patients accessing specialist hospice day and community services at two Marie Curie clinical services in England and Scotland. Single-case experimental designs are appropriate for this kind of study because they allow for highly controlled intervention delivery [30] and a patient-centred, in-depth analytic strategy. We use them here to facilitate process modelling and accurate intervention development [31] in line with MRC guidance for the development of complex interventions [32]. They are idiographic in nature and also allow for the detailed examination of effects through quantification of outcome and process variables [33].

The primary aim of a single-case experimental design is to indicate that any observed change in outcome results from the application of the intervention. Single-case designs are commonly used in psychological intervention research [34] and have proven utility in cancer research [35]. As an intervention study, this is a phase I ‘modelling’ study [32] that will lead on to a later feasibility trial of the developed, manualised, intervention. As such, delivery of the current study will be under optimal conditions (i.e. by trained psychologists/psychotherapists) to ensure high levels of fidelity and content quality, in addition to creating a therapeutically and ethically safe environment for participants and intervention facilitators. Establishing proof of principle in this way is a vital first step in fully developing and evaluation a complex intervention [32].

### Intervention: rationale and development

Previous research, including pilot work conducted by our team [18–20, 23], demonstrates that ACT is a psychological intervention that is both relevant and acceptable to people with cancer. Intervention content for this specific study was initially developed through a thorough review of literature describing common challenges and psychological problems faced by cancer patients at the stage of advanced cancer/palliative care. The literature describes common themes around: loss of control [36]; fear, understanding and acceptance [37]; rapid loss of independence, shifting relationship dynamics and the importance of balancing life tasks against limited time available [38].

Alongside this conceptual review, we used general ACT intervention guidance [e.g. 39, 40], resources available on the Association for Contextual Behavioural Science website (www.contextualscience.org), and ACT intervention development expertise within the study research team to plan treatment strategies and methods likely to be effective in promoting psychological flexibility: effective living, in the presence of the kinds of psychological responses described in the literature above. The intervention was developed by two ACT experts (DG and NHW) and then iteratively refined in consultation with the broader research group and our study patient and stakeholder reference group (including two cancer patients, a family carer and a healthcare professional working in a hospice setting).

The intervention manual consists of five sessions (see Table 1). Each is designed for one-to-one, face-to-face delivery, and to last approximately 45 to 60 min per session. At this early stage of intervention development work, we have designed the intervention to be delivered by an experienced therapist with either a doctoral level clinical psychology qualification or a Masters level CBT qualification (or equivalent) conferring active British Association of Cognitive and Behavioural Therapy accreditation. Intervention facilitators are not required to have prior experience of working in a palliative care setting; during induction, they will undertake the standard hospice induction which includes observation and orientation in the hospice. Previous training and experience in delivering ACT-based intervention is desirable, though not essential for this study.

**Table 1:** Intervention components

Module	Purpose	Elements
A	Assessment and engagement	Warmth, empathy, positive regard. History taking, typical responses to transition, beginning baseline monitoring and introducing measurement protocol and concepts.
B	Workability	Review of typical responses and greater contact with the consequences, linking ineffective strategies with control, avoidance and cognitive fusion.
C	Awareness	Teaching awareness skills, linking to greater behavioural choice, mindfulness exercises, 5 senses experience, mindful eating a raisin, 10-min mindfulness audio exercise given for homework.
D	Openness	Demonstrating the greater effectiveness of willingness to have difficult thoughts and feelings and at the same time, stepping back from such inner experiences. Using leaves on the stream exercise, singing negative thoughts, speaking negative thoughts in a funny voice, perspective taking around thoughts, kick your buts exercise, ‘I’m having the thought that…’ exercise.
E	Engagement	Linking behavioural effectiveness with desired outcomes and qualities of actions, in order to live with purpose and meaning in the end stage of life. Concept of values, and actions, sweetspot exercise, the compass metaphor, generating hierarchies of difficult actions.
F	Review and ending (1 month follow up)	Review of progress after 4 weeks of no treatment, barriers to practice, anticipation of future challenges and how open, aware and engagement skills could be used, behavioural rehearsal of effective responses, commitments to next steps. Ending contact.

In addition to local hospice induction, training will be provided by the intervention authors (NHW and DG) through the use of self-study materials, simulated video role-play, and practice role-play opportunities. The level of training provided will be tailored dependent on the prior experience and skills of the individuals appointed to this role. Before delivery of the intervention to study participants, therapists will undertake a competency assessment where their delivery of the intervention (with a simulated patient) will be video-recorded and assessed against a previously validated ACT rating scale [39]. Therapists are required to score at least 90 (corresponding to ‘average competence’) before delivering the intervention in the clinical setting. Where this level of competency is not achieved, further training will be provided. There will be one therapist at each clinical site to ensure continuity for participants recruited and consistency of delivery between participants for added methodological quality. Clinical supervision will be provided by an existing member of the team who is suitably qualified for this role (JSt).

Module A takes the majority of the first intervention session. Session two then covers module B plus one of the ACT-specific modules (C, D or E) in an order determined by the therapist to best meet the needs and priorities identified and negotiated with the participant in session one. Sessions 3 and 4 cover the remaining module content (C, D or E), with module F delivered at 1-month follow-up. The level of flexibility inherent in the design will allow for greater understanding of delivery differences between participants and a more refined understanding of which elements produce what kinds of changes in outcome and process variables.

Each week, participants are provided with homework exercises to build skills. These include sheets to help prompt mindfulness activities, defusion exercises, values creation and committed actions based on identified values. Homework exercises also include short audio recordings to prompt mindfulness, values contact, and defusion skills. Summary sheets of the key themes of each session are provided to participants at the end of each session to be used as aide-memoire; a modified version of this is also provided to be given to any family members or carers who have questions about the intervention content. The family/carer summary sheets are for information only rather than intending to be used for personal benefit.

### Participants: eligibility criteria and referral into the study

People over 16 years of age who have been told that they have an incurable cancer diagnosis and who are referred to specialist hospice day or community services at two hospice sites (one in England and one in Scotland) will be identified by hospice-based community nursing teams and invited to participate (see Table 2). Eligible participants must have a life expectancy of 4 months or more and, although limited to those affected by cancer, are not restricted to any particular cancer type. Seven to ten participants will be recruited from each recruitment site with the aim of completing the intervention with fourteen participants in total.

**Table 2:** Protocol for recruitment and intervention delivery

1.	Identification of eligible participants by hospice team.
2.	Clinical teams to introduce study during routine visit to hospice/day service within 2 weeks of referral into service: information sheet provided and consent given to pass contact details to research team.
3.	Consent form returned to either clinical team or posted directly to research team. If returned to clinical team, to be posted to research team.
4.	Researcher has weekly contact with each clinical team and ensures maintenance of consent log each site. Maintains corresponding participant database.
5.	When consent form received by researcher, researcher makes contact with participant to: • Provide opportunity for further questions • Establish time frame for intervention delivery and • Arrange intervention sessions with psychotherapist for relevant site. Copy of consent form sent to clinical team to be placed in patient medical record.
6.	Researcher maintains log of sessions for therapist.
7.	Intervention session 1 (45–60 min) covering the assessment and engagement components, completion of baseline assessments, and preparing the participant to undertake the daily diary recordings. One week completion of baseline daily diary recordings.
8.	After 1 week completion of daily diaries (baseline), intervention session 2 (30–45 min) and completion of weekly questionnaires (15 min). Daily diary data collection continues.
9.	Intervention session 3 (30–45 min) and completion of weekly questionnaires (15 min). Daily diary data collection continues.
10.	Intervention session 4 (30–45 min) and completion of weekly questionnaires (15 min). Daily diary data collection continues.
11.	Intervention session 5 (follow-up session) approximately 4 weeks later (30–45 min) and completion of weekly questionnaires (15 min). Information sheet and consent form given for interview. Daily diary data collection end with this session providing 9 weeks of data in total.
12.	Interview consent form received by research team. Copy provided to clinical team for patient medical record.
13.	Interview conducted by telephone 2 weeks after the end of the intervention (30 min).

### Information and consent processes

Community nurses who undertake an initial assessment on referral into the hospice services will provide an overview of the study and provide a study pack which includes a patient information sheet, information for family members and a study consent form. Participants will be given time to consider the study invitation and then, if interested, will be asked to provide consent to be contacted by the research team. At this point, the researcher will contact participants by telephone and (i) explain the study in greater detail, covering intervention aims and assessment; (ii) give participants an opportunity to ask questions; (iii) explain practicalities such as session frequency, location and transport; and (iv) arrange a first appointment. Written informed consent will be taken as part of the first session with the therapist.

### Procedure

Ethical approval has been obtained from an NHS Research Ethics Committee (IRAS Project ID: 239683), in addition to relevant approvals from the research governance committees at Marie Curie Hospice Liverpool and Marie Curie Hospice Edinburgh. These two hospices have been selected pragmatically due to proximity to the collaborating University research sites; however, they afford a comparison between delivery across (a) two UK countries and (b) settings both with (Edinburgh) and without (Liverpool) a dedicated in-house hospice research lead.

Intervention sessions will take place at the hospice and last between 45 and 60 min. During the assessment interview, participants will complete baseline measures described below. These measures are repeated at the start of each subsequent intervention session. Daily process and QoL data will be gathered using either the smart-phone based app (Personal Analytics Companion (PACO) [40]) or a paper and pencil booklet equivalent. Completion of daily diaries will be monitored by the research team, and where three consecutive recordings are missed, the researcher will contact the participant by telephone to discuss barriers to completion. In the case of non-app-based diary data collection, we will not be able to undertake such regular checking (this can happen only at face-to-face meetings with the therapist for these participants), and thus, smartphone data collection methods will be strongly encouraged.

During the final intervention session, the therapist will explain the purpose of the follow-up qualitative interview, providing both written information and an opportunity to answer any questions that participants might have about this. Consent will be collected at that time by the therapist. Two weeks later, participants will be contacted by the researcher. Interviews will be conducted on a one-to-one basis either in the hospice (face-to-face) or via telephone. Interviews will be recorded for later transcription and analysis. The interview schedule is designed to help participants to express their experiences of taking part, acceptability of intervention content and perceived effectiveness of the intervention and its components. In addition, the interview will also ask about caregivers’ experiences during the intervention.

On completion of the study, participants will be provided with a study debrief sheet, including information about getting additional support. Procedures for dealing with any distress due to the interview have been carefully considered and pathways into further support outlined and agreed with the relevant clinical care teams.

Once the study has been completed in each clinical site, we will invite all staff members involved with the study (recruitment, site-specific study management etc.) to focus groups where we will undertake discussions and data collection on any challenges and barriers that they experienced during the study. We will also seek their opinions about acceptability, effectiveness and impact on the patient and their family and suggestions for how the study design should be improved for later trials.

### Data collection

Weekly self-report questionnaires will be administered throughout the intervention to assess changes in quality of life (our primary outcome), distress (secondary outcome) and ACT intervention processes (as potential mediating variables).

Daily diaries will be used to attempt more sensitive and specific measurement of process change. Daily recordings are known to produce more valid and accurate results than retrospective summary reports [41]. Participants will have the choice to access these daily recordings either via a smartphone app or paper versions to ensure ease of access. The use of a smartphone app (PACO [40]) for daily data collection is relatively novel within palliative care trials, and so, this pilot data will allow us to conclude whether this is a feasible alternative to paper-based data collection methods for similar work.

Data will also be collected on the number of patients approached about the study, the number then recruited, attrition, and reasons for non-eligibility in order to inform later feasibility and effectiveness trials.

### Primary outcome

#### Functional Assessment of Chronic Illness Therapy—palliative care (weekly) [42]

The FACIT-pal measures the functional quality of life in palliative care. The scale is comprised of five domains: physical well-being (7 items), social/family well-being (7 items), emotional well-being (6 items), functional well-being (7 items) and additional concerns (19 items). Each item is measured on a Likert scale of 0 (not at all) to 4 (very much). Cronbach’s alpha has previously been reported at .90 for the full 27-item scale and .78 to .87 for sub-scales, suggesting strong internal consistency [43].

### Secondary outcomes

#### Distress thermometer (weekly) [44]

The DT is a single-item measure of distress on a scale from 0 (no distress) to 10 (extreme distress). It has been validated as a worldwide measure of distress in people affected by cancer [45].

#### Comprehensive assessment of Acceptance and Commitment Therapy processes (weekly) [46]

The CompACT is a measure of the postulated mechanism of action and process of ACT. It consists of 23 items grouped into three sub-scales (openness to experience, behavioural awareness and valued action) which map closely onto our three core intervention sessions (modules C, D and E). Items are scored on a 7-point Likert scale, ranging from 0 (strongly disagree) to 6 (strongly agree). Psychometric properties of the CompACT have been shown to be adequate [46].

#### Brief Acceptance Measure (daily) [47]

The BAM is a three-item measure of psychological flexibility, specifically designed for use in single-case experimental designs and similar daily diary studies. Participants rate the previous 24 h on each of three dimensions of openness, awareness and engagement in valued activities on a 1 to 10 numeric scale. Scales are anchored at either end with indicative statements (e.g. 1 = struggling with thoughts, feelings and physical sensations versus 10 = open to thoughts, feelings and physical sensations). The BAM total score has an alpha of .71 and correlates strongly with the CompACT (*r = .*57, *p* < .001). The BAM is also sensitive to brief interventions designed to increase psychological flexibility in healthy adults.

#### Single-item QoL (daily)

A single quality of life item will be used to measure overall health. This is an item in which participants rate their health on a scale from 0 (worst imaginable health state) to 100 (best imaginable health state).

#### Feasibility outcomes

Although this study is not designed as a phase II feasibility study, relevant data will be collected on the number of patients screened for eligibility, number patients potentially eligible, number of patients recruited, and percentages of consented participants who complete the intervention and follow-up. Percentage drop-out, deaths during the study, and missing data (i.e. how much of the self-report questionnaires were completed by participants) will be monitored and recorded throughout the study. This information, together with the pilot acceptability data, will be used to inform the next phase of our research—a full feasibility study—by clarifying whether this is a potentially appropriate setting and timepoint in the patient pathway to deliver a psychological intervention. Undertaking full feasibility testing at this stage is inappropriate given that we do not yet have fully developed, acceptable, intervention content.

### Sample size

Potentially high rates of attrition need to be considered in any palliative care research when determining the sample size [51]. We aim to recruit 14 patients who complete the full intervention. Given a high probability of attrition, we expect to recruit approximately 20 patients allowing for such attrition. This sample size is more than sufficient for a single-case research design [48, 49] which purposely recruits only small samples given their idiographic focus. All participants who complete the intervention will be invited to participate in the qualitative interview.

### Data analysis

#### Quantitative data

Analysis of daily diary data (BAM and QoL single-item) will include visual analysis, calculation of stability envelope, between-phase mean level change and the percentage non-overlapping data, as is standard to single-case methodology [48]. Advances in statistical analysis of single-case experimental design data now allow researchers to calculate *p* values and effect sizes from single-case data, and to aggregate these across single cases, using metanalytic methods [49]; such analyses will be undertaken where data permit their use. These data will provide tentative and preliminary indications of effectiveness and will be reported with 95% confidence intervals where relevant. Weekly questionnaire data will be analysed with visual analysis and level change [50].

#### Management of missing data

We assume that some participants will be unable to complete the weekly and daily self-report questionnaires at certain times during the study period. Given that the weekly questionnaires will be filled out immediately before each session and administered by the therapist, we anticipate no participants missing any weekly questionnaires. However, it is likely that participants will miss opportunities to record the daily measures. Members of the hospice team will be made aware if a participant misses three or more daily recordings in a row in order to determine if the reason is due to health deterioration prior to the research team following up with the participant directly to discuss any technical issues in recording this data.

#### Qualitative data

Follow-up interviews with participants will be audio-recorded, transcribed verbatim and analysed using a framework analysis approach [52]. Framework analysis is particularly well suited for applied health research that aims to answer specific questions within a limited time frame [53]. It allows for the analysis of data thematically whilst undertaking an exploratory analysis of the dataset. Staff focus groups will be analysed using this same analytic approach.

### Manual revision

Alongside data collection from participants, and the focus groups with hospice staff, we will also seek expert peer-review of the manual. The intervention manual will be circulated to experts within our own professional networks, including (a) ACT experts, (b) clinical psychologists who do not identify primarily as ACT-oriented, (c) cancer/palliative care nurses and (d) palliative care specialists. We will aim to seek feedback from at least five expert reviewers who are independent from our project team.

Peer-review feedback will be triangulated with the quantitative and qualitative data from the empirical part of this study to inform a revised version of the intervention manual. The revised manual will be worked on initially by the original two authors (DG and NHW) and then presented to the broader research group and the study patient and stakeholder reference group for comments before finalising at the end of the study.

## Discussion

There is a growing appreciation for the need for holistic support for people with cancer that cannot be cured who are transitioning into specialist palliative care settings; this needs to include evidence-based psychological care [22]. Our work aims to build on a small existing literature by pilot testing a novel, brief and manualised ACT-based intervention to improve psychological support for this population. Our work will determine whether or not the intervention is feasible and acceptable in this setting and will provide a foundation for subsequent research to test intervention efficacy in potentially more scalable formats, such as delivery by nurses and allied health professionals, in either group or individual settings.

The use of a mixed-method, single-case design will provide both quantitative and narrative data that will indicate potential efficacy and mechanism of the intervention and also allow an in-depth analysis of patient experience. This is a rare design choice in psychosocial oncology and palliative care research, and we intend for our study to provide an example of how this approach can be well-suited to phase I intervention development and modelling work [32].

If this development phase proves successful, the next phase in our programme of work will be to feasibility test the manualised intervention using potentially more cost-effective means of delivery and more conventional, group-based randomised controlled designs. To allow this, we will use data collected in this development study to revise the content of the intervention manual. This pilot work will generate acceptability data, and information on practical challenges and potential barriers to uptake and retention, to ensure maximised likelihood of success in these follow-on trials.

Key to the success of this study will be our embedded approach of involving people affected by cancer [54]. In addition to a patient representative as a grant co-applicant and member of the Project Management Group (SM), we have recruited a patient and stakeholder reference group who will input on intervention content, pilot trial design, analysis of data and refinement of the intervention manual on study completion. This will be based on more extensive feedback from patient participants in the study who take part in the embedded qualitative interviews.

## Dissemination plan

A primary output from this study will be a published, open-access, delivery manual for the intervention. Although this will not have been robustly efficacy tested, we expect the data from this study may be sufficient to allow health care professionals to choose whether or not they wish to use this immediately. Findings from this study will be published in peer-review journal articles and at a range of conferences, including palliative care, psychosocial oncology and psychological intervention audiences for maximised impact. A lay summary will be available for participants and their families and to be circulated to key stakeholder organisations.

## Conclusions

This paper reports a study protocol and so we are limited in the conclusions that we can make at this stage. However, the forthcoming results will have high importance for both theory and palliative care literatures. Regarding theoretical implications, this study will expand our knowledge of how the ACT framework can be adapted and applied to people with cancer that cannot be cured, as they transition into palliative care services: this is a previously unexplored application of the ACT intervention framework. There is a high need for data-driven research into how we can best support the psychological and supportive care needs of people transitioning into care: our study is the first step in developing a novel intervention to fill this crucial research and service-delivery gap.

## Data Availability

Current datasets are not available as data is currently being collected. Once data are fully gathered, the datasets used and/or analysed during this study are available from the corresponding author on reasonable request.

## Abbreviations

ACT: Acceptance and Commitment Therapy
